# Factors associated with primary transmission of multidrug-resistant tuberculosis compared with healthy controls in Henan Province, China

**DOI:** 10.1186/s40249-015-0045-1

**Published:** 2015-03-24

**Authors:** Wei-Bin Li, Yan-Qiu Zhang, Jin Xing, Zhen-Ya Ma, Ya-Hong Qu, Xin-Xu Li

**Affiliations:** Institute for Tuberculosis Control and Prevention of Kaifeng prefecture, Henan Province Kaifeng, 475000 People’s Republic of China; Henan Center for Disease Control and Prevention, Zhengzhou, 450016 PRC; National Center for Tuberculosis Control and Prevention, Chinese Center for Disease Control and Prevention, 155 Changbai Road, Changping District Beijing, 102206 PRC

**Keywords:** Tuberculosis, Primary MDR-TB, Factors, Matched case–control, China

## Abstract

**Background:**

It is estimated that there are about 74,000 primary multidrug-resistant tuberculosis (MDR-TB) patients per year according to the prevalence of MDR-TB of 5.7% among new TB patients in China. Thus, the risks of primary transmission of MDR-TB require further attention. This study aimed to identify the factors associated with primary transmission of MDR-TB in Henan province, where the number of new TB patients is ranked second highest in China.

**Methods:**

A 1:1 matched case–control study was conducted in Henan, China. Cases were primary MDR-TB patients who were individually matched with a healthy control without TB from the same neighborhood. The study was conducted from July 2013 to June 2014. Both case and control were matched by age (±5 years) and sex. Conditional logistic regression was used to compute adjusted odds ratios (AORs) with corresponding 95% confidence intervals (CIs) for risk factors associated with primary MDR-TB.

**Results:**

For the study, 146 pairs of participants were recruited. The final multivariable logistic regression model disclosed that after adjusting for age and sex, primary MDR-TB cases were more likely to be single (AOR, 5.4; 95% CI, 1.4–20.7), earn an annual income of ≤ 12,000 yuan (RMB) (AOR, 9.9; 95% CI, 2.0–48.1), experience more life pressure/stress (AOR, 10.8; 95% CI, 2.8–41.5), not be medically insured (AOR, 50.1; 95% CI, 8.2–306.8), and suffer from diabetes, cardiovascular disease or other respiratory diseases, or cancer (AOR, 57.1; 95% CI, 8.6–424.2).

**Conclusions:**

In order to control primary transmission of MDR-TB in China, we recommend that improving the social support, living standards and medical security of the lower social class become a priority.

**Electronic supplementary material:**

The online version of this article (doi:10.1186/s40249-015-0045-1) contains supplementary material, which is available to authorized users.

## Multilingual abstracts

Please see Additional file 1 for translations of the abstract into the six official working languages of the United Nations.

## Background

Multidrug-resistant tuberculosis (MDR-TB), defined as TB caused by strains of *Mycobacterium tuberculosis* that is resistant to at least isoniazid (INH) and rifampicin (RFP), is strongly related to irregular or weak treatment of TB [1-3]. The rates of MDR-TB in previously treated TB cases are significantly higher than in newly diagnosed TB cases [4-6]. Compared with acquired MDR-TB that is developed in the course of TB treatment, it is easy to neglect primary MDR-TB (defined as having no previous history of TB alongside primary infection with resistant bacteria) for MDR-TB control because of the lower rates associated with it. The proportion of MDR-TB is 5.7% in new TB cases and 25.6% in previously treated TB cases in China [7,8]. However, lower rates don’t necessarily equate with fewer cases. About 870,000 new cases and 54,000 retreatment cases were notified in China in 2010, which suggests that the estimated number of primary MDR-TB cases is higher than that of acquired MDR-TB cases.

Nosocomial transmission of MDR-TB among human immunodeficiency virus (HIV) infected patients and health workers as well as household transmission of MDR-TB have been investigated since the 1990s [9-11]. In recent years, the extensive person-to-person transmission of MDR-TB has been of concern in many countries. A study in Papua New Guinea showed transmission of MDR-TB, whilst another study in Spain suggested that MDR-TB in a low-incidence region showed a high rate of transmission [12,13]. China also documented that transmission of MDR-TB strains was a serious concern in big cities and some rural areas [14,15]. The transmission of MDR-TB in the general population is a huge public health challenge for MDR-TB control. Thus, prompt, effective, and appropriate actions to interrupt the transmission must be urgently taken.

However, research on independent or dependent determinants of primary MDR-TB is scanty, and research on the risk factors in a representative data associated with primary MDR-TB is scarce in China. Although a study reported a high rate of primary MDR-TB in a general population with no identifiable risk factors for MDR-TB [16], more studies on primary MDR-TB are needed to verify this. In 2012, the number of new TB cases in Henan province ranked second highest in China. Therefore, we aimed to explore the risk factors possibly associated with primary transmission of MDR-TB in Henan, such as sociodemographic characteristics, daily behaviors, and medical experiences, and provide insights into effective primary MDR-TB control, i.e. interrupting transmission of MDR-TB in the general population in China.

## Methods

Between July 2013 and June 2014, 146 primary MDR-TB cases were selected in Henan. The cases were confirmed by five prefecture-level TB dispensaries that implemented the MDR-TB control programs of the Global Fund, and by a prefecture-level TB dispensary that implemented the MDR-TB control program of the Bill & Melinda Gates Foundation. All MDR-TB cases were identified by the laboratories of these prefecture-level TB dispensaries, using sputum culture and drug susceptibility testing (DST), with the proportion method for INH, RFP, ethambutol, and streptomycin, following the Guidelines for Surveillance of Drug Resistance in Tuberculosis (Second Edition) published by the World Health Organization (WHO) [17]. All laboratories of these prefecture-level TB dispensaries were accredited to diagnose MDR-TB by the TB reference laboratory of the Henan Center for Disease Control and Prevention (Henan CDC).

Each primary MDR-TB case was individually matched with a healthy control using the local population management system. The candidates for each control were selected from the same neighborhood of each case i.e. living in the nearest village or community. The age (±5 years) and gender of the control was matched to its corresponding case. The matched control was included if he or she did not have confirmed TB or any other pulmonary disease, as according to the diagnostic criteria of the National Tuberculosis Control Program in China [18], and if he or she had the most similar matching conditions to the case. All participants were systematically tested for HIV.

A structured questionnaire was developed and pre-tested before interviews with study participants began (see Additional file 2). This study was evaluated and approved by the Ethics Review Committees (ERCs) of both the Henan CDC and the China CDC. Before the interviews, all participants were asked for their written informed consent. The questionnaire covered sociodemographic characteristics, daily behaviors, and medical experiences of primary MDR-TB cases in the year prior to them becoming patients, and of healthy controls in the same year. Office workers/jobs that require mental skills and manual labor workers are referred to as skilled and unskilled workers, respectively, in this study. All the interviews were conducted in participants’ homes, rooms of local CDCs, or TB dispensaries, as chosen by the participants. The matched participants were interviewed on the same day by the same interviewer. Privacy was ensured while interviews were being conducted.

A conditional logistic regression model was used to account for the matched case–control study design. All analyses were performed using the SAS statistical package (version 9.2, SAS Institute, Inc., Cary, NC). Univariable analysis was done for computing unadjusted matched odds ratios (ORs) and their 95% confidence intervals (CIs). The level of significance was assessed by the Wald *χ*^2^ test. Variables with *p*-values < 0.05 were entered into a multivariable conditional logistic regression model to simultaneously examine their independent effects (adjusted ORs, AORs) through stepwise deletion of variables. The final model was obtained until all predictors left had *p*-values < 0.05.

## Results

As shown in Table 1, 146 primary MDR-TB cases and matched controls were enrolled in the study. The mean ages of both groups were 48 ± 17 years old and 48 ± 16 years old, respectively, and 66.7% were males. Compared with controls, primary MDR-TB cases were more likely to have an education level of middle school or below (43.1% versus 17.6%) and be single (33.3% versus 17.6%). There were similar proportions of employment between primary MDR-TB cases and controls: 19.6% and 13.7% were unemployed, 31.4% and 39.2% were skilled or unskilled workers, 25.5% and 23.5% were migrant workers, and 23.5% and 23.5% were farmers, respectively. Compared with controls, primary MDR-TB cases were more likely to earn less than 12,000 yuan (RMB) per year (82.4% versus 51.0%) and live in a room smaller than 40 m^2^ (68.6% versus 41.2%).

**Table 1:** **Sociodemographic characteristics of primary MDR-TB cases and controls, sex and age (±5 years) matched**

***Variables***		***Primary MDR-TB cases***	***Controls***	***Wald χ*** ^***2***^ ***value***	***p-value***	***COR (95%CI)***
		***n (%)***	***n (%)***			
**Total (N)**		146 (100.00)	146 (100.00)	NA	NA	NA
***Sex***	Male	97 (66.67)	97 (66.67)	NA	NA	NA
	Female	49 (33.33)	49 (33.33)	NA	NA	NA
***Age (years)***	(mean ± standard deviation)	48 ± 17	48 ± 16	NA	NA	NA
	[median (min, max)]	48 (18, 79)	49 (18, 77)	NA	NA	NA
***Education***	Middle school and above	83 (56.86)	120 (82.35)			1.00
	Illiterate or elementary school	63 (43.14)	26 (17.65)	6.78	0.0092	4.25 (1.43–12.63)
***Single***	No	97 (66.67)	120 (82.35)			1.00
	Yes	49 (33.33)	26 (17.65)	4.32	0.0377	5.00 (1.10–22.81)
***Employment***	Unemployed	29 (19.61)	20 (13.73)			1.00
	Skilled or unskilled worker	46 (31.37)	57 (39.22)	1.02	0.3132	0.53 (0.15–1.82)
	Migrant worker	37 (25.49)	34 (23.53)	0.17	0.6763	0.76 (0.21–2.79)
	Farmer	34 (23.53)	34 (23.53)	0.35	0.5554	0.62 (0.13–3.01)
***Annual income (RMB)***	>12,000 yuan	26 (17.65)	72 (49.02)			1.00
	≤12,000 yuan	120 (82.35)	74 (50.98)	8.83	0.0030	6.33 (1.87–21.40)
***Area of residence***	Urban	57 (39.22)	77 (52.94)			1.00
	Rural	89 (60.78)	69 (47.06)	2.24	0.1344	2.00 (0.81–4.96)
***Size of living space***	≥40 m^2^	46 (31.37)	86 (58.82)			1.00
	<40 m^2^	100 (68.63)	80 (41.18)	6.34	0.0188	3.00 (1.28–7.06)
***Floor in building of living space***	Second floor and above	40 (27.45)	83 (56.86)			
	Below the second floor	106 (72.55)	63 (43.14)	7.69	0.0056	4.00 (1.50–10.66)

NOTE: COR: crude odds ratio; CI: confidence interval; NA: not applicable.

The univariable ORs with 95% CIs for potential risk factors were evaluated for their association with primary MDR-TB (see Tables 1, 2, and 3). Compared with controls, risk factors significantly associated with primary MDR-TB were illiteracy or primary school education (OR, 4.25; 95% CI, 1.43–12.63), being single (OR, 5.00; 95% CI, 1.10–22.81), an annual income ≤ 12,000 yuan (RMB) (OR, 6.33; 95% CI, 1.87–21.40), living space < 40 m^2^ (OR, 3.00; 95% CI, 1.28–7.06), living below the second floor (OR, 4.00; 95% CI, 1.50–10.66), body mass index ≤ 20 (OR, 3.75; 95% CI, 1.23–11.30), eating fruit once in intervals of > 7 days (OR, 3.50; 95% CI, 1.15–10.63), exercising once in intervals of ≥ 2 days (OR, 4.33; 95% CI, 1.24–15.20), more life pressure/stress (OR, 4.00; 95% CI, 1.50–10.66), not being medically insured (OR, 8.00; 95% CI, 1.84–34.79), and suffering from diabetes, cardiovascular disease, other respiratory diseases, or cancer (OR, 12.00; 95% CI, 1.56–92.27).

**Table 2:** **Daily behaviors of primary MDR-TB cases and controls, sex and age (±5 years) matched**

***Variables***		***Primary MDR-TB cases***	***Controls***	***Wald χ*** ^***2***^ ***value***	***p-value***	***COR (95% CI)***
		***n (%)***	***n (%)***			
**Total (N)**		146 (100.00)	146 (100.00)	NA	NA	NA
***Body mass index***	>20	92 (62.75)	123 (84.31)			1.00
	≤20	54 (37.25)	23 (15.69)	5.52	0.0188	3.75 (1.23–11.30)
***Interval of days of eating meat***	≤1	92 (62.75)	103 (70.59)			1.00
	>1	54 (37.25)	43 (29.41)	0.61	0.4346	1.36 (0.63–2.97)
***Interval of days of eating coarse food grain***	≤2	80 (54.90)	86 (58.82)			1.00
	>2	66 (45.10)	60 (41.18)	0.28	0.5943	1.33 (0.46–3.84)
***Interval of days of eating fruit***	≤7	94 (64.71)	123 (84.31)			1.00
	>7	52 (35.29)	23 (15.69)	4.88	0.0271	3.50 (1.15–10.63)
***Hours of sleep each night***	>7	89 (60.78)	86 (58.82)			1.00
	≤7	57 (39.22)	60 (41.18)	0.04	0.8475	0.93 (0.44–1.98)
***Interval of days of doing physical exercise***	<2	20 (13.73)	49 (33.33)			1.00
	≥2	126 (86.27)	97 (66.67)	5.24	0.0221	4.33 (1.24–15.20)
***More life pressure/stress***	No	86 (58.82)	129 (88.24)			1.00
	Yes	60 (41.18)	17 (11.76)	7.69	0.0056	4.00 (1.50–10.66)
***Interval of weeks of going to crowded fields***	>1	92 (62.75)	103 (70.59)			1.00
	≤1	54 (37.25)	43 (29.41)	0.98	0.3226	1.67 (0.61–4.59)
***Never smoking***	Yes	92 (62.75)	83 (56.86)			1.00
	No	54 (37.25)	63 (43.14)	0.47	0.4931	0.73 (0.29–1.81)
***Never drinking alcohol***	Yes	72 (49.02)	66 (45.10)			1.00
	No	74 (50.98)	80 (54.90)	0.22	0.6380	0.80 (0.32–2.03)

NOTE: COR: crude odds ratio; CI: confidence interval; NA: not applicable.

**Table 3:** **Medical experiences of primary MDR-TB cases and controls, sex and age (±5 years) matched**

***Variables***		***Primary MDR-TB cases***	***Controls***	***Wald χ*** ^***2***^ ***value***	***p-value***	***COR (95% CI)***
		***n (%)***	***n (%)***			
**Total (N)**		146 (100.00)	146 (100.00)	NA	NA	NA
***Medically insured***	Yes	97 (66.67)	137 (94.12)			1.00
	No	49 (33.33)	9 (5.88)	7.69	0.0056	8.00 (1.84–34.79)
***Being vaccinated with BCG vaccine***	Yes	77 (52.94)	92 (62.75)			1.00
	No	69 (47.06)	54 (37.25)	1.28	0.2571	1.71 (0.68–4.35)
***Ever visiting hospitals***	No	106 (72.55)	94 (64.71)			1.00
	Yes	40 (27.45)	52 (35.29)	0.79	0.3744	0.67 (0.27–1.63)
***Suffering from diabetes, cardiovascular disease, other respiratory diseases, or cancer***	No	109 (74.51)	140 (96.08)			1.00
	Yes	37 (25.49)	6 (3.92)	5.70	0.0170	12.00 (1.56–92.27)
***Possible exposure to TB cases***	No	80 (54.90)	86 (58.82)			1.00
	Yes	66 (45.10)	60 (41.18)	0.13	0.7156	1.14 (0.56–2.34)

NOTE: COR: crude odds ratio; CI: confidence interval; NA: not applicable

As shown in Table 4, risk factors independently associated with primary MDR-TB were being single (AOR, 5.37; 95% CI, 1.39–20.67), earning an annual income ≤ 12,000 yuan (RMB) (AOR, 9.93; 95% CI, 2.05–48.11), having more life pressure/stress (AOR, 10.80; 95% CI, 2.81–41.52), not being medically insured (AOR, 50.07; 95% CI, 8.17–306.81), and suffering from diabetes, cardiovascular disease, other respiratory diseases, or cancer (AOR, 57.05; 95% CI, 8.59–424.17).

**Table 4:** **Multivariable conditional logistic regression model showing risk factors independently associated with primary MDR-TB**

***Variables***	***Wald χ*** ^***2***^ ***value***	***p-value***	***AOR (95% CI)***
Single (No = 0, Yes = 1)	5.96	0.0146	5.37 (1.39–20.67)
Annual income (RMB) (>12,000 yuan = 0, ≤ 12,000 yuan = 1)	8.13	0.0044	9.93 (2.05–48.11)
More life pressure/stress (No = 0, Yes = 1)	12.00	0.0005	10.80 (2.81–41.52)
Being medically insured (Yes = 0, No = 1)	8.76	0.0031	50.07 (8.17–306.81)
Suffering from diabetes, cardiovascular disease, other respiratory diseases, or cancer (No = 0, Yes = 1)	6.46	0.0110	57.05 (8.59–424.17)

NOTE: AOR: adjusted odds ratio; CI, confidence interval.

## Discussion

Findings from this study showed that being single, earning a low income, having mental stress, lacking medical insurance, and suffering from a chronic debilitating disease were potential risk factors associated with primary MDR-TB. However, risk factors of nosocomial transmission and close contact were not found. Although many risk factors associated with MDR-TB were found in many studies [1-3], only one study researched risk factors associated with primary MDR-TB, with no identifiable risk factors reported prior to this [16]. Therefore, our study has value and provided a timely report on the risk factors of primary MDR-TB.

Marital status, as one kind of sociodemographic characteristics, is often related to diseases, including TB. Studies in Henan and Hong Kong highlighted that marital status was independently associated with TB [19,20]. Being single was a risk factor for primary MDR-TB in this study, which was in agreement with many other studies that showed that single persons were more likely to develop TB [21,22]. Although there isn’t an identifiable biological relationship between marital status and TB, single persons are more likely to lack social support or be involved in high-risk behaviors, such as alcohol consumption, which potentially leaves them exposed to higher risks of infection of TB or MDR-TB than those with other marital statuses [23,24].

Like marital status, income levels are also interrelated with disease and health. In 2011, the WHO reported that most low-income countries had high TB and MDR-TB rates [8]. A study in China suggested that low household economic conditions were associated with TB [19], and studies in Turkey and China found that a low socioeconomic status or poverty were associated with MDR-TB [3,25]. In this study, low income was also a risk factor associated with primary MDR-TB. Consequently, we infer that it is easy for TB patients in low-income communities to acquire MDR-TB because there is a lack of good medical services. With the increase of acquired MDR-TB patients in low-income communities, the general population has higher chances of contracting MDR-TB. In other words, inhabitants of low-income communities are more likely to become primary MDR-TB patients. This finding also suggests that primary transmission of MDR-TB is not extensive in the general population and still confined to low-income communities.

This study found that those who had high life pressures/stress were more likely to be primary MDR-TB patients, which was not shown in previous studies on MDR-TB [1-6]. China’s development comes an increasing pressure that can trigger higher levels of mental stress in both low-income and high-income groups. It is known that mental stress can affect health by inducing immunologic and cardiovascular changes [26,27]. If mental stress develops into a severe mental illness, it might become a risk factor associated with TB [28]. Therefore, we speculate that mental stress induced by heavy life pressures can suppress the body’s immune system and reduce the body’s resistance, giving way to disease, such as infections of the upper respiratory tract [29], thus creating opportunities for the transmission of MDR-TB.

Our study also found that a lack of medical insurance was another risk factor associated with primary MDR-TB. Many studies in China showed that not having medical insurance influenced a patient’s access to TB care, due to the high cost of drugs, or led to failure of anti-TB treatment [30,31]. This might also indicate that medical insurance affects medical-seeking behaviors and medical services, and perhaps TB is representative of many diseases impacted by medical insurance. Most people without medical insurance in China probably delay medical-seeking behaviors, which worsen conditions in the body and unavoidably erode its defense mechanisms. Therefore, it is easy to understand that those who cannot afford medical insurance are more likely to be infected with MDR-TB.

Among the risk factors independently associated with primary MDR-TB in our study, suffering from diabetes, cardiovascular disease, other respiratory diseases, or cancer was another biologic risk factor. All these diseases fall into the chronic debilitating disease category. Previous studies confirmed that chronic debilitating diseases, such as diabetes, chronic respiratory diseases, and solid-organ malignancy, were risk factors for TB [32-34]. Another study reported that the impaired immunity in type 2 diabetes possibly increased susceptibility to infection with resistant strains of TB [35], which is consistent with our finding. Chronic debilitating diseases that can impair or suppress the body’s immunity potentially increase the risks for primary MDR-TB.

The independent risk factors for primary MDR-TB in our study are related to sociodemographic characteristics, daily behaviors, and medical experiences. This points to the fact that the risk factors associated with TB in general are not too dissimilar to risk factors associated with MDR-TB, perhaps because primary MDR-TB is one of the subgroups of TB. Therefore, it seems that usual TB control measures would also be applicable to primary MDR-TB patients. Despite this, the findings of this study can provide specific clues for monitoring new TB cases with high risk factors for MDR-TB, and even identifying MDR-TB transmission hotspots in the general population [36]. The study can also provide specific clues for improving the integrated, comprehensive, interventional response packages that are used to control primary transmission of MDR-TB. An effective way of doing this is enhancing the social support, living standards, and medical security of the lower social class in China.

Before summing up the main points of this study, we must outline its limitations. We collected data of primary MDR-TB cases and healthy controls in previous years, so recall biases were unavoidable and potentially affected the results. Data such as annual income, size of living space, and interval of days when certain foods were eaten might be inaccurate as it was collected by self-reporting. Additionally, there were some primary MDR-TB cases who were from rural areas adjacent to urban areas, but as we could not find appropriate healthy controls from their areas (according to their age and gender), we had to select the controls from the urban areas. Despite these limitations, our study provided some useful information for understanding risk factors of primary MDR-TB in China.

## Conclusion

This matched case–control study identified risk factors associated with primary MDR-TB in Henan province, China. These included being single, having a low income, being mentally stressed, not being medically insured, and suffering from a chronic debilitating disease. On the contrary, other risk factors such as nosocomial transmission and close contact were not found. Perhaps it can be inferred from these findings that, currently, primary transmission of MDR-TB is not confined to nosocomial transmission and close contact, but is confined to low-income groups in the general population. These findings can be helpful to identify new TB cases with high risk factors for primary MDR-TB, and to improve the comprehensive, interventional response packages that are used to control primary MDR-TB by improving social support, living standards, and medical security of lower social groups in China.

## Additional files

Additional file 1: Multilingual abstracts in the six official working languages of the United Nations. Additional file 2: The structured questionnaire.
