# Supplementary material for: Factors associated with primary transmission of multidrug-resistant tuberculosis compared with healthy controls in Henan Province, China
Source: Infect Dis Poverty. 2015 Mar 24;4:14. doi: 10.1186/s40249-015-0045-1 (PMC4371877; doi:10.1186/s40249-015-0045-1)
Supplement: Additional file 1: — Multilingual abstracts in the six official working languages of the United Nations. [file 40249_2015_45_MOESM1_ESM.pdf]

Translation of the abstract into the six official working languages of the United Nations

العوامل المرتبطة بانتقال مرض السُل (الدَرَن) البدئي المقاوم لعديد العقاقير بالمقارنة مع أمثلة صحّية (غير مصابة) في إقليم هينان بالصين.

ويي-بين لي ، يان-كيو زهانج ، جين كسينج ، زهين-يا ما ، يا-هونج كو ، كسين-كسو لي.

ملخّص:

خلفية: من المقدَّر أن هناك سنوياً حوال 74 ألف مريض بالسُل البدئي المقاوم لعديد العقاقير وفقاً لانتشار ذلك النوع من السُل بنسبة 5.7% وسط المرضى الجُدد به في الصين. وعليه ، فإن خطر الانتقال للسل البدئي المقاوم العديد العقاقير يتطلّب مزيداً من اليقظة. وهذه الدراسة تهدف إلى التعرّف على المُنبئات المرتبطات بانتقال السُل (الدَرَن) البدئي المقاوم لعديد العقاقير في إقليم هينان حيث يأتي ترتيب عدد المرضى الجدد بالسل ثاني أعلى عدد في الصين.

الوسائل: أُجريت دِراسَةُ حالاتٍ وشَّواهِد (دِراسَةٌ استرجاعية/استِعادِيَّة لحالات انفرادية مقارنة بحالات ضابطة) بنسبة 1:1 في هينان بالصين. كانت الحالات هي لمرضى سُل (دَرَن) بدئي مقاوم لعديد العقاقير قُورنت كل على حدة بمثال صحّي بدون سُل (دَرَن) من ذات الجوار. أُجريت الدراسة من يوليو (تموز) 2013 إلى يونيو (حُزيران) 2014 ؛ وتمّت المقارنة بين كل من الحالة المرضية والمثال الصحّي من حيث العمر (± 5 سنوات) والجنس (ذكر/أنثى). لقد استُخدِم النكوص المنطقي المشروط (CLR) (نوع من النماذج التصنيفية الإحصائية ذات الاحتمال ، والتي تُستخدَم في تقدير متغيّرات نموذج ما للاستجابة النوعية.الكيفية) وذلك لإحصاء نسبة الترجيح الموازَنة (AORs) مع فترات إسْتِئْمان/إطْمِئْنان (CIs) مقابلة بنسبة 95% من عوامل الخطر المرتبطة بالسُل (الدَرَن) البدئي المقاوم لعديد العقاقير.

النتائج: تم تجنيد 146 مثنى (زوج) مشارك للدراسة ، وأظهر النموذج النهائي للنكوص المنطقي المشروط (CLR) متعدد المتغيّرات أنه بعد الموازنة بالنسبة إلى العمر والجنس ، أن حالات السُل (الدَرَن) المقاوم لعديد العقاقير كانت فردية في الاحتمال الغالب (نسبة الترجيح الموازَنة AOR ، 5.4 ؛ فترة إسْتِئْمان/إطْمِئْنان CI مقابلة بنسبة 95% ، 1.4 – 20.7) ، وأنّها تكسب دخلاً سنوياً يساوي أو أقل من 12 ألف يوان (RMB) ، (نسبة الترجيح الموازَنة AOR ، 9.9 ؛ فترة إسْتِئْمان/إطْمِئْنان CI مقابلة بنسبة 95% ، 2.0 – 48.1) ، وتعاني من ضغط/توتّر حياتي أكبر (نسبة الترجيح الموازَنة AOR ، 10.8 ؛ فترة إسْتِئْمان/إطْمِئْنان CI مقابلة بنسبة 95% ، 2.8 – 41.5) ، وغير مُؤمَّن عليها طبّياً (نسبة الترجيح الموازَنة AOR ، 50.1 ؛ فترة إسْتِئْمان/إطْمِئْنان CI مقابلة بنسبة 95% ، 8.2 – 360.8) ، وتعاني من السكّري ، أو مرض ضيق الشرايين أو أمراض أخرى في الجهاز التنفّسي ، أو السرطان (نسبة الترجيح الموازَنة AOR ، 57.1 ؛ فترة إسْتِئْمان/إطْمِئْنان CI مقابلة بنسبة 95% ، 8.6 – 424.2).

الاستنتاجات: للسيطرة على انتقال السُل (الدَرَن) البدئي المقاوم لعديد العقاقير في الصين فإننا نوصي بأن يصبح تحسين الدعم الاجتماعي ، ومستويات المعيشة ، والتأمين الصحي للطبقة الاجتماعية الشعبية أولوية من الأولويات.

Translated from English version into Arabic by Lotfi Abdolhaleem, through

TRANSLATORS WITHOUT BORDERS

中国河南省以健康人群作为对照的原发性耐多药肺结核的危险因素研究

李卫彬，张艳秋，邢进，马振亚，屈亚虹，李新旭

摘要

引言：中国新发肺结核患者中有 5.7%为耐多药肺结核患者。按照这个比例估计，中国每年约有 74,000 例原发性耐多药肺结核患者。因此，耐多药肺结核在人群间的原发传播风险需要引起进一步关注。河南省每年登记的新发肺结核患者数量位居中国第二位，因此，为了解原发性耐多药肺结核在人群中传播的危险因素，本研究选在河南省开展。

方法：按照 1:1 个体匹配的病例对照研究方法，每例原发性耐多药肺结核患者匹配一名健康对照，要求健康对照未患过肺结核，与患者来自同一社区或村庄，具有相似的年龄（±5 岁）和相同的性别。该研究于 2013 年 7 月至 2014 年 6 月在中国河南省开展。条件 logistic 回归模型用于计算与原发性耐多药肺结核有关的危险因素的调整 OR 值（AOR）及其 95%可信区间（CI）。
结果：共有 146 对研究对象纳入到本研究中。多因素 logistic 回归模型显示，对年龄和性别进行调整后，单身（AOR，5.4；95% CI，1.4–20.7）、个人年收入≤12,000 元人民币（AOR，9.9；95% CI，2.0–48.1）、感觉生活压力较大（AOR，10.8；95% CI，2.8–41.5）、没有医疗保险（AOR，50.1；95% CI，8.2–306.8）和患过糖尿病、心血管病、其他呼吸道疾病或癌症（AOR，57.1；95% CI，8.6–424.2）是罹患原发性耐多药肺结核的危险因素。
结论：为了在中国有效控制耐多药肺结核在人群间的原发传播，我们建议把改进社会底层群体的社会支持、生活条件和医疗保障作为优先措施。

Translated from English version into Chinese by LI Xin-xu

Facteurs associés avec la transmission primaire de la tuberculose multirésistante comparée avec des sujets témoins en bonne santé de la province du Henan, en Chine.

Wei-Bin Li, Yan-Qiu Zhang, Jin Xing, Zhen-Ya Ma, Ya-Hong Qu, Xin-Xu Li

Résumé

Contexte: On estime qu'il y a environ chaque année en Chine 74 000 personnes atteintes de tuberculose multirésistante (MDR-TB), selon la prévalence de la MDR-TB qui est de 5.7% chez les nouveaux patients tuberculeux. Désormais, les risques de transmission primaire de la MDR-TB nécessitent une attention particulière. Cette étude avait pour objet de déterminer les facteurs de prédiction associés avec la transmission primaire de la MDR-TB dans la province du Henan, classée deuxième en Chine en ce qui concerne le nombre de nouveaux patients atteints de tuberculose.
Méthodes: Une analyse appariée 1:1 de cas témoins a été réalisée au Henan, en Chine. Les cas dont il s'agissait étaient principalement des patients atteints de la MDR-TB appariés de manière individuelle avec des sujets du même voisinage non atteints de tuberculose. Cette étude a été effectuée de juillet 2013 à juin 2014. On a ensuite apparié les membres du groupe témoin aux sujets sains selon leur age (+/- 5 ans) et leur sexe. Les données ont été analysées par régression logistique conditionnelle pour calculer les ratios de probabilité révisés (AORs) avec des intervalles de confiance à 95% (CIs) pour des facteurs de risques associés à la MDR-TB primaire.
Résultats: Pour cette étude, 146 paires de participants ont été recrutés. Le modèle définitif de regression logistique à variables multiples a révélé qu'après les ajustements liés à l'age et au sexe, les sujets atteints de MDR-TB étaient plus susceptibles d'être célibataires, (AOR, 5.4; 95% CI, 1.4-20.7), d'avoir un revenu annuel égal ou inférieur à 12000 yuan (RMB) (AOR, 9.9; 95% CI, 2.0-48.1), de ressentir plus de pression/stress dans leur vie, (AOR, 10.8; 95% CI, 2.8-41.5) d'être sans assurance médicale (AOR, 50.1; 95% CI, 8.2-306.8) et de souffrir de diabète, de maladie cardio vasculaire ou d'une autre maladie respiratoire, ou de cancer (AOR, 57.1; 95% CI, 8.6-424.2).
Conclusions: Afin de contr ôler la transmission primaire de la MDR-TB en Chine, nous recommandons, pour la classe sociale défavorisée, que l'amélioration du soutien social, du niveau de vie et de la sécurité en ce qui concerne les soins médicaux deviennent une priorité.

Translated from English version into French by chantaldefrance, through

Факторы, связанные с первичными путями передачи туберкулёза с множественной лекарственной устойчивостью, в сравнении со здоровыми контролями в провинции Хэнань, Китай

Вэй-Бин Ли, Ян-Чью Жанг, Джин Шинг, Жэн-Я Ма, Я-Хонг Чжу, Шин-Шю Ли

Абстракт

Базовая проблематика: Согласно коэффициенту распространенности туберкулёза с множественной лекарственной устойчивостью (МЛУ ТБ) в 5.7% от всех новых случаев заболевания туберкулезом в Китае, считается, что число пациентов с первичным МЛУ ТБ равняется 74 000 в год. Таким образом, риск передачи МЛУ ТБ первичными путями заслуживает больше внимания. Это исследование направлено на определение прогностических факторов, связанных с первичными путями передачи МЛУ ТБ в провинции Хэнань, находящейся на втором месте по числу новых случаев заболевания туберкулезом в Китае.

Методы: Исследование методом случай-контроль 1:1 было проведено в провинции Хэнань, Китай. Случаями послужили пациенты с первичным МЛУ ТБ, с индивидуально подобранными здоровыми контролями без туберкулеза из того же района. Исследование проводилось в июле 2013 – июле 2014. Случаи и контроли подбирались по возрасту (± 5 лет) и полу. Для вычисления скорректированного отношения шансов (СОШ) с соответственными 95%-ми доверительными интервалами (ДИ) для факторов риска, связанных с первичным МЛУ ТБ, была использована условная логистическая регрессия.

Результаты: К исследованию было привлечено 146 участников. Окончательная модель многопараметрической логистической регрессии показала, что после корректировки с учетом возраста и пола, случаи заболевания первичным МЛУ ТБ с большей долей вероятности наблюдались среди следующих участников: холостые/незамужние (СОШ, 5.4; 95% ДИ, 1.4 – 20.7), с годовым доходом ≤ 12 000 юаней (RMB) (СОШ, 9.9; 95% ДИ, 2.0–48.1), испытывающие больше стресса в жизненной ситуации (СОШ, 10.8; 95% ДИ, 2.8–41.5), без медицинской страховки (СОШ, 50.1; 95% ДИ, 8.2–306.8) и страдающие диабетом, сердечно-сосудистыми заболеваниями, другими заболеваниями дыхательной системы или раком (СОШ, 57.1; 95% ДИ, 8.6–424.2).

Заключение: Для профилактики передачи МЛУ ТБ первичными путями, мы рекомендуем, в первую очередь, повышение качества социальной поддержки, уровня жизни и медицинского страхования низших слоев общества.

Translated from English version into Russian by Nurangiz Khodzharova, through

Factores asociados con la transmisión primaria de la tuberculosis multidrogorresistente en comparación con

casos control sanos en la provincia de Henán, China.

Wei-Bin Li, Yan-Qiu Zhang, Jin Xing, Zhen-Ya Ma, Ya-Hong Qu, Xin-Xu Li

Resumen

Antecedentes: Se estima que existen alrededor de 74 000 pacientes por año con tuberculosis multidrogorresistente (TB-MDR), de acuerdo con el valor de prevalencia de la TB-MDR del 5.7 % entre los nuevos pacientes con TB en China. Por este motivo, es necesario prestar más atención a los riesgos de transmisión primaria de la TB-MDR. El objetivo de este estudio fue identificar los indicadores de la transmisión primaria de TB-MDR en la provincia de Henán, donde el número de nuevos pacientes de TB es el segundo más alto de China.

Metodología: A 1: Se realizó un estudio de casos y controles en Henán, China. Los casos fueron pacientes primarios de TB-MDR que estaban emparejados con un control sano sin TB, proveniente del mismo vecindario. El estudio se realizó entre julio de 2013 y junio de 2014. Los casos y los controles fueron emparejados por edad (±5 años) y sexo. Se utilizó una regresión logística condicional para calcular el cociente de probabilidades ajustado (AOR, por sus siglas en inglés) con los intervalos de confianza (IC) del 95 % correspondientes para factores de riesgo asociados con la TB-MDR primaria.

Resultados: para el estudio se reclutaron 146 pares de participantes. El modelo final de regresión logística multivariada reveló que luego de ajustar por edad y sexo, los casos primarios de TB-MDR tenían más tendencia a ser solteros (AOR, 5.4; 95% IC, 1.4-20.7), a tener un ingreso anual ≤ 12,000 yuan (RMB) (AOR, 9.9; 95% IC, 2.0–48.1), a tener más presiones/estrés cotidianos (AOR, 10.8; 95% IC, 2.8–41.5), a no tener seguro médico (AOR, 50.1; 95% IC, 8.2–306.8), y a sufrir de diabetes, enfermedades cardiovasculares u otras enfermedades respiratorias, o cáncer (AOR, 57.1; 95% IC, 8.6–424.2).

Conclusiones: A fin de controlar la transmisión primaria de la TB-MDR en China, recomendamos priorizar la ayuda social, el nivel de vida y la seguridad médica de las clases sociales más bajas.

Translated from English version into Spanish by Nora Glembocki, through

TRANSLATORS
WITHOUT BORDERS
