# Supplementary material for: Factors associated with primary transmission of multidrug-resistant tuberculosis compared with healthy controls in Henan Province, China
Source: Infect Dis Poverty. 2015 Mar 24;4:14. doi: 10.1186/s40249-015-0045-1 (PMC4371877; doi:10.1186/s40249-015-0045-1)
Supplement: Additional file 2: — The structured questionnaire. [file 40249_2015_45_MOESM2_ESM.doc]

**Questionnaire**

For: 0 Primary MDR-TB case 0 Healthy control

Survey ID: 0 0 0

1. Sex: (1) Male (2)Female (3) Others (Please specify: )

2. Birthdate: (YYYY/MM/DD)

| For the question 3 to 5, the information are collected from case history of primary MDR-TB case and annual medical report of healthy control. |
| --- |

3. For primary MDR-TB case, he or she was diagnosed as primary MDR-TB in the year (YYYY). For whether primary MDR-TB case or healthy control, all the information **in the year before primary MDR-TB was diagnosed** are collected. (For example, if a primary MDR-TB case was diagnosed as primary MDR-TB in 2011, for whether primary MDR-TB case or healthy control, all the information in 2010 are collected.)

4. Weight: Kg

5. Height: cm

6. Marital status: (1) Single (including unmarried, divorced and spouses loss)

(2) Cohabitation (3) Married (4) Others (Please specify: )

7. Occupation: (1) Staff (2) Worker (3) Migrant worker (4) Farmer

(5) Unemployed (6) Others (Please specify: )

8. Annual per capita income (RMB): Yuan

9. Region of residence: (1) Urban area (2) Rural area

(3) Others (Please specify: )

10. Per capital living space: m^2^

11. Inhabitancy floor: (1) Basement (2) First floor (3) Second floor and above

(4) Others (Please specify: )

12. Interval days of eating meats once: days

13. Interval days of eating coarse food grain once: days (Coarse food grain refers to corn, millet, sorghum, oat, buckwheat, bean, and so on)

14. Interval days of eating fruits once: days

15. Hours of sleep every day: hours

16. Interval days of physical exercise once: days (Physical exercise refers to running, swimming, dancing, playing all kinds of sports balls, and so on.)

17. Feeling higher life pressure: (1) Yes (2) No (3) Others (Please specify: )

18. Interval weeks of going to crowded fields once: weeks (Crowded field refers to internet bar, song and dance halls, billiard room, gymnasium, chess room, foot bath room, sauna, cinema, and so on.)

19. Smoking: (1) Yes (2) No only in this year (3) Never

(4) Others (Please specify: )

20. Drinking alcohol: (1) Yes (2) No only in this year (3) Never

(4) Others (Please specify: )

21. Having medical insurance: (1) Yes (2) No (3) Others (Please specify: )

22. Ever vaccinating BCG vaccine: (1) Yes (2) No

(3) Others (Please specify: )

23. Ever visiting hospitals: (1) Yes (2) No (3) Others (Please specify: )

24. Suffering from some diseases: (1) Yes (Please specify: ) (2) No

25. Possible exposure to TB cases: (1) Yes (2) No

(3) Others (Please specify: )
